# Effect of TiO_2_ nanotubes with TiCl_4_ treatment on the photoelectrode of dye-sensitized solar cells

**DOI:** 10.1186/1556-276X-7-579

**Published:** 2012-10-23

**Authors:** Teen-Hang Meen, Yi-Ting Jhuo, Shi-Mian Chao, Nung-Yi Lin, Liang-Wen Ji, Jenn-Kai Tsai, Tien-Chuan Wu, Wen-Ray Chen, Walter Water, Chien-Jung Huang

**Affiliations:** 1 Department of Electronic Engineering, National Formosa University, Yunlin, 632, Taiwan; 2 Department of Electrical Engineering, Hsiuping University of Science and Technology, Taichung, 412, Taiwan; 3 Institute of Electro-Optical and Materials Science, National Formosa University, Yunlin, 632, Taiwan; 4 Department of Applied Physics, National University of Kaohsiung, Kaohsiung, 811, Taiwan

**Keywords:** TiO_2_ nanotube arrays, dye-sensitized solar cells, TiCl_4_ treatment

## Abstract

In this study, we used the electrochemical anodization to prepare TiO_2_ nanotube arrays and applied them on the photoelectrode of dye-sensitized solar cells. In the field emission scanning electron microscopy analysis, the lengths of TiO_2_ nanotube arrays prepared by electrochemical anodization can be obtained with approximately 10 to 30 μm. After titanium tetrachloride (TiCl_4_) treatment, the walls of TiO_2_ nanotubes were coated with TiO_2_ nanoparticles. XRD patterns showed that the oxygen-annealed TiO_2_ nanotubes have a better anatase phase. The conversion efficiency with different lengths of TiO_2_ nanotube photoelectrodes is 3.21%, 4.35%, and 4.34% with 10, 20, and 30 μm, respectively. After TiCl_4_ treatment, the efficiency of TiO_2_ nanotube photoelectrode for dye-sensitized solar cell can be improved up to 6.58%. In the analysis of electrochemical impedance spectroscopy, the value of *R*_k_ (charge transfer resistance related to recombination of electrons) decreases from 26.1 to 17.4 Ω when TiO_2_ nanotubes were treated with TiCl_4_. These results indicate that TiO_2_ nanotubes treated with TiCl_4_ can increase the surface area of TiO_2_ nanotubes, resulting in the increase of dye adsorption and have great help for the increase of the conversion efficiency of DSSCs.

## Background

Dye-sensitized solar cells (DSSCs) have received considerable attention lately because they are cost-effective and environmentally friendly with efficiencies comparable to those of the traditional silicon-based cells [1]. Generally, granular titanium dioxide powder is commonly used in dye-sensitized solar cell light anode structure. The sol–gel method is used to produce porous film structure, but small pores form between particles of the transmission path of clutter, resulting in a more dye adsorption capacity and low clutter of electron transfer path. The path is too long and will make the leakage current and the probability of electron recombination, thus affecting the overall conversion efficiency of solar cells. The titanium dioxide nano-tubular structure of high surface area and large aspect ratio can be beneficial to the dye adsorption, and more rules of order can be reduced when the electron and hole in the transmission probability of recombination. TiO_2_ nanotubes have been synthesized by various methods including hydrothermal method [2], seeded growth [3], template-assisted deposition [4], and anodization [5]. Especially, anodization is a relatively simple method for synthesizing large-area and self-organized TiO_2_ nanotube arrays [6-8]. In this paper, we used the electrochemical anodization to prepare TiO_2_ nanotubes arrays with different thickness and applied them on the photoelectrode of dye-sensitized solar cells. The TiO_2_ nanotubes and solar cells were investigated by field-emission scanning electron microscopy, X-ray diffraction (XRD), *IV* characteristic analyses, and electrochemical impedance spectroscopy (EIS) to study the effect of titanium tetrachloride (TiCl_4_) treatment on the photoelectrode of TiO_2_ nanotubes for dye sensitized solar cells.

## Methods

In this study, the growth of nanotubes was anodized on Ti foils (purity of 99.6%, thickness of 0.2 mm) by constant current at 15 mA in the ethylene glycol solution containing 0.3 wt.% NH_4_F and 2 vol.% deionized water kept at 20°C. The anodized TiO_2_ nanotubes were annealed in oxygen at 450°C for 60 min. For the treatment of TiCl_4_, TiO_2_ nanotubes were immersed in 0.2 M TiCl_4_ solution for 1 h and annealed in air at 350°C for 30 min. Pt counter electrodes were prepared by coating with a drop of H_2_PtCl_6_ solution and heating at 400°C for 15 min [9]. To adsorb N3 dye, TiO_2_ nanotubes were immersed in 3×10^−4^ M solution containing N3 dye and ethyl alcohol at 45°C for 8 h in the oven. The working electrodes were then rinsed with ethanol. Electrolyte solution is adopted from Everlight Chemical Industrial Corporation (ESE-20). The electrode was assembled into a sandwich-type open cell using platinum plate as a counter electrode. Both electrodes were spaced by a kind of polymer films. The thickness was 60 μm, and the size of TiO_2_ working electrode was 0.25 cm^2^ (0.5 ×0.5 cm). The surface morphology of the TiO_2_ nanotubes was observed by scanning field emission electron microscopy. Structural analysis was carried out by powder X-ray diffraction (XRD). The ultraviolet–visible absorption spectrum of the TiO_2_ electrodes was observed by a UV–vis spectrophotometer. The current–voltage characteristics and impedance of samples were measured by Keithley 2400 source meter (Keithley Instruments Inc., Cleveland, OH, USA), and EIS was determined under simulated sunlight with white light intensity, *P*_L_ = 100 mW/cm^2^.

## Results and discussion

**Figure 1:**
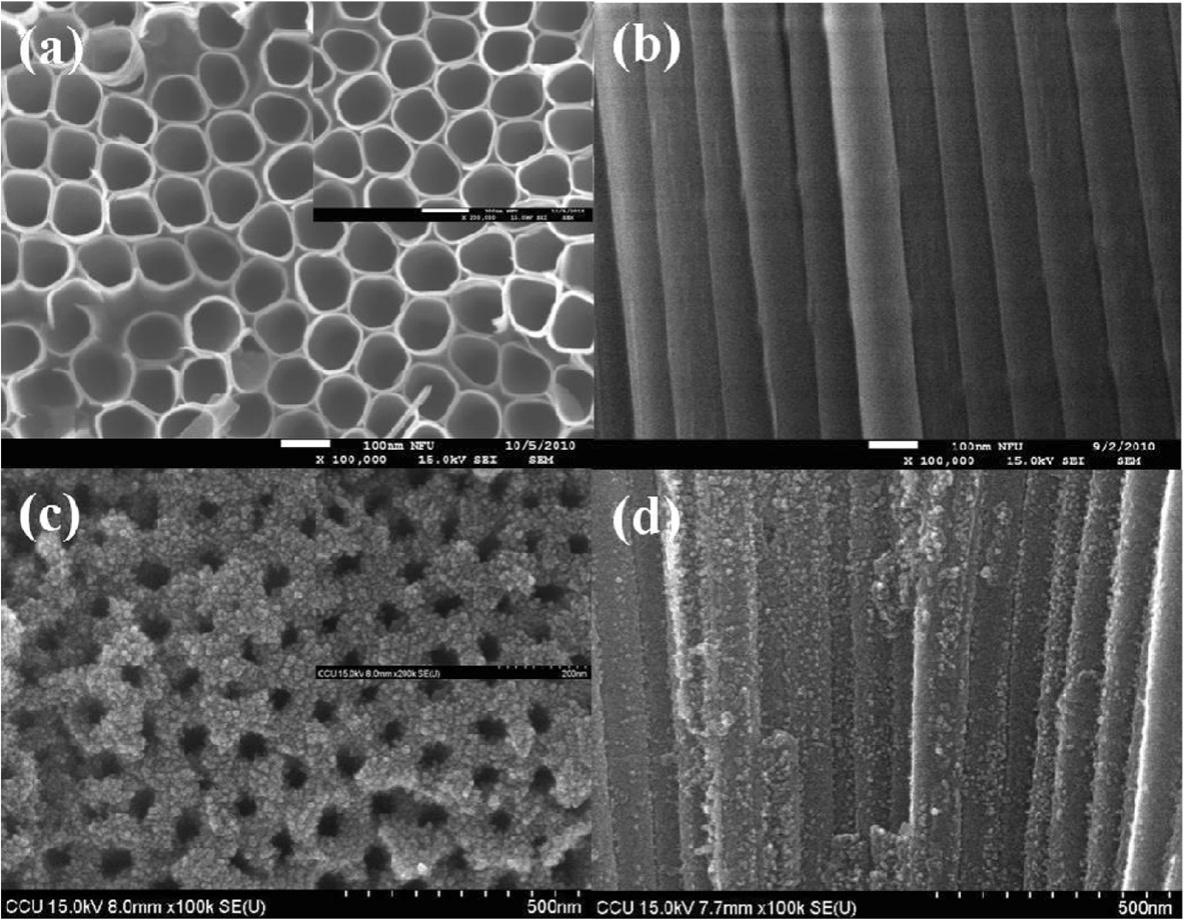
**SEM images of TiO**_**2 **_**nanotubes.** (**a**) Top view and (**b**) side view before TiCl_4_ treatment, and (**c**) top view and (**d**) side view after TiCl_4_ treatment.

**Figure 2:**
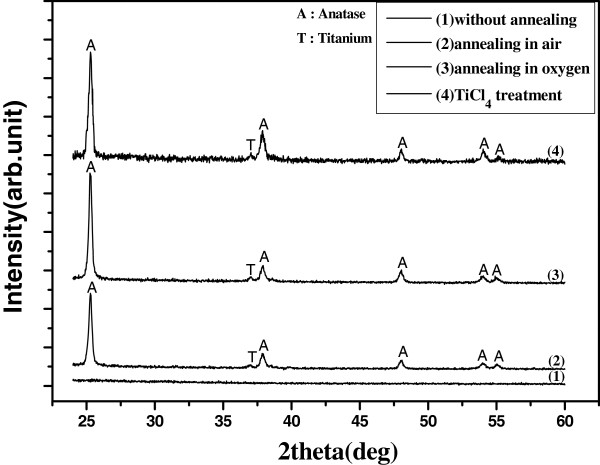
**XRD patterns of TiO**_**2 **_**nanotubes.**

**Figure 3:**
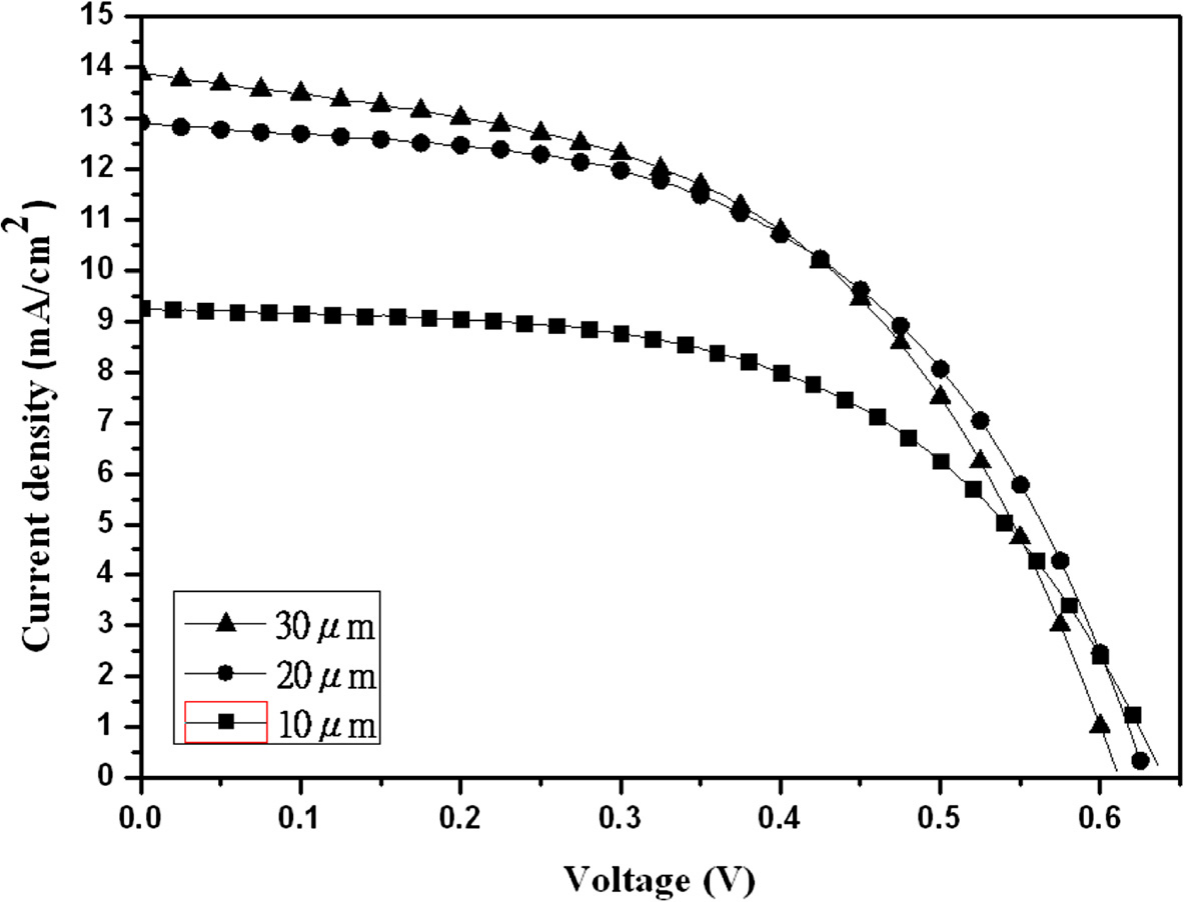
**The *I *- *V *curves of DSSCs with different lengths of TiO**_**2 **_**nanotubes.**

**Figure 4:**
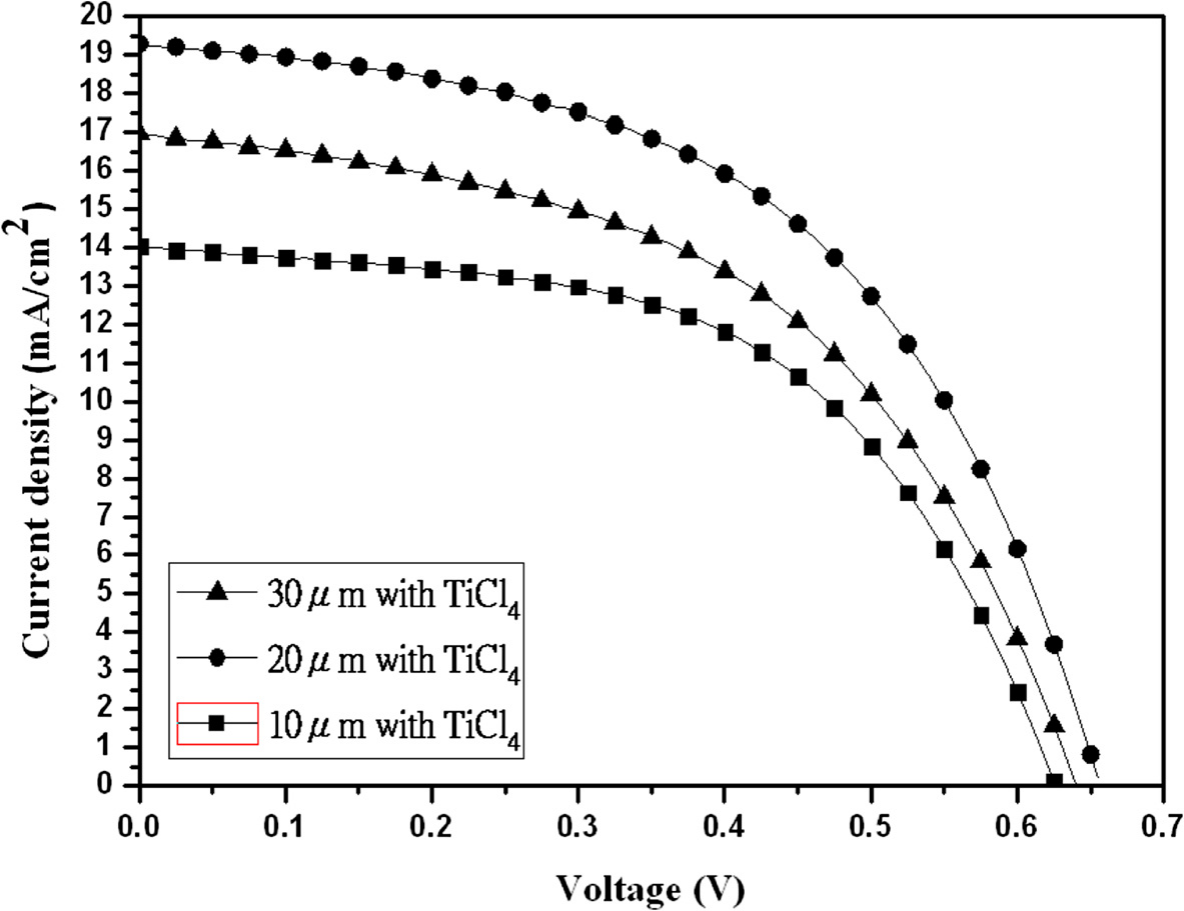
**The *I *- *V *curves of DSSCs with different lengths of TiO**_**2 **_**nanotubes after TiCl**_**4 **_**treatment.**

Figure 1 shows the SEM images of the TiO_2_ nanotubes before and after TiCl_4_ treatment. Clearly, after the samples were treated with TiCl_4_, the walls of TiO_2_ nanotubes were coated with TiO_2_ nanoparticles, which could increase the surface area of TiO_2_. In order to explore the impact of annealing gas on the properties of TiO_2_ nanotubes, the samples were carried out with XRD characterization. XRD patterns of TiO_2_ nanotubes are shown in Figure 2. It is found that the as-formed TiO_2_ nanotubes are amorphous and are converted to anatase after annealing. The oxygen annealed TiO_2_ nanotubes have a better anatase phase than that annealed in air. After the treatment of TiCl_4_, TiO_2_ nanotubes also show a good anatase phase. Figure 3 shows the current–voltage characteristics of DSSCs with the electrodes of different lengths of TiO_2_ nanotubes without TiCl_4_ treatment. The parameters for the short-circuit current density (*J*_sc_), the open circuit potential (*V*_oc_), the fill factor, and the overall conversion efficiency (*η*) are listed in Table 1. From the results of Figure 3 and Table 1, it is found that the best conversion efficiency of DSSCs is 4.35%, while the length of TiO_2_ nanotubes is 20 μm. The result of conversion efficiency is quite higher than the previous reports [10-12]. This may be due to the length of TiO_2_ nanotubes in this study, which is quite longer than those of the previous reports. It is advantage to adsorb N3 dye on the TiO_2_ nanotubes. Figure 4 shows the current–voltage characteristics of DSSCs with the electrodes of different lengths of TiO_2_ nanotubes after TiCl_4_ treatment. The parameters for the *J*_sc_, the *V*_oc_, the fill factor, and the *η* are listed in Table 2. From the results of Figure 4 and Table 2, it is found that the best conversion efficiency of DSSCs can be improved up to 6.58%, while the length of TiO_2_ nanotubes is 20 μm.

**Table 1:** **The parameters of current–voltage characteristics for DSSCs with different lengths of TiO**_**2**_**nanotubes**

**Sample (μm)**	***V***_**oc**_**(V)**	***J***_**sc**_**(mA/cm**^**2**^**)**	***V***_**m**_**(V)**	***I***_**m**_**(mA/cm**^**2**^**)**	**FF (%)**	***η*(%)**
10	0.63	9.56	0.44	7.29	53.63	3.21
20	0.63	12.92	0.43	10.12	53.61	4.35
30	0.61	13.89	0.41	10.59	51.11	4.34

**Table 2:** **The parameters of current–voltage characteristics for DSSCs with different lengths of TiO**_**2 **_**nanotubes after TiCl**_**4 **_**treatment**

**Sample (μm) with TiCl**_**4**_	***V***_**oc**_**(V)**	***J***_**sc**_**(mA/cm**^**2**^**)**	***V***_**m**_**(V)**	***I***_**m**_**(mA/cm**^**2**^**)**	**FF (%)**	***η*(%)**
10	0.63	14.03	0.44	11.06	54.77	4.81
20	0.66	19.27	0.46	14.30	52.00	6.58
30	0.64	16.97	0.43	12.67	50.15	5.45

**Figure 5:**
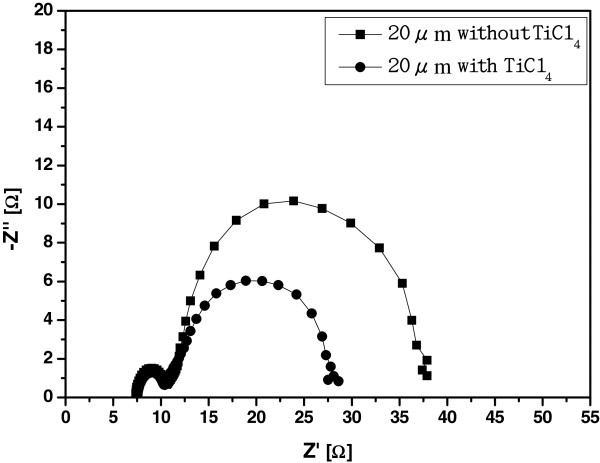
**Spectra of EIS for the dye-sensitized solar cells with and without TiCl**_**4 **_**treatment.**

In order to study the effect of TiCl_4_ treatment on the transport properties of TiO_2_ nanotubes, the analysis of EIS for TiO_2_ nanotubes has been investigated. Figure 5 shows the spectra of EIS for the dye-sensitized solar cells with and without TiCl_4_ treatment. The simulation of equivalent circuit is referred to the previous reports [13-15]. The parameter *R*_k_, which is represented by charge transfer resistance related to recombination of electrons, is also listed in Table 3. The value of *R*_k_ decreases from 26.1 to 17.4 Ω after TiCl_4_ treatment. These results indicate that the effect of TiCl_4_ treatment on TiO_2_ nanotubes can increase the surface area of TiO_2_ and the adsorption of N3 dye, resulting in better transport properties of TiO_2_ nanotubes and the improvement of conversion efficiency for DSSCs.

**Table 3:** **The parameters of EIS calculated from Figure**5** for dye-sensitized solar cells with and without TiCl**_**4 **_**treatment**

**Sample**	***Κ***_**eff**_**(s**^**−1**^**)**	***τ***_**eff**_**(s)**	***R***_**s**_**(Ω)**	***R***_**pt**_**(Ω)**	***R***_**k**_**(Ω)**	***η*(%)**
20 μm without TiCl_4_	0.97	1.03	7.35	3.35	26.1	4.35
20 μm with TiCl_4_	1.44	0.69	7.54	2.86	17.4	6.58

## Conclusions

In summary, we prepared TiO_2_ nanotube arrays by electrochemical anodization to apply on the electrode of dye-sensitized solar cell. After TiCl_4_ treatment, the walls of TiO_2_ nanotubes were coated with TiO_2_ nanoparticles. It can increase the surface area of TiO_2_ and the adsorption of N3 dye, resulting in better transport properties of TiO_2_ nanotubes and the improvement of conversion efficiency of DSSCs.

## Abbreviations

DSSCs: Dye-sensitized solar cells; EIS: Electrochemical impedance spectroscopy; *I*-*V* characteristics: Current–voltage characteristics; *J*_sc_: Short-circuit current density; *η*: Overall conversion efficiency; TiCl_4_: Titanium tetrachloride; *V*_oc_: Open circuit potential; XRD: X-ray diffraction.

## Competing interests

The authors declare that they have no competing interests.

## Authors' contributions

THM wrote this manuscript. YTJ and NYL carried out the preparation of samples. SMC and LWJ carried out the XRD measurements. JKT and TCW carried out the *I*-*V* measurements. WRC, WW, and CJH carried out the EIS measurements. All authors read and approved the final manuscript.
